# The action of microbial collagenases in dentinal matrix degradation in root caries and potential strategies for its management: a comprehensive state-of-the-art review^*^

**DOI:** 10.1590/1678-7757-2024-0013

**Published:** 2024-05-20

**Authors:** Cecília de Brito BARBOSA, Isabela MONICI SILVA, Naile DAME-TEIXEIRA

**Affiliations:** 1 Universidade de Brasília Faculdade de Ciências da Saúde Departamento de Odontologia Brasília Brasil Universidade de Brasília, Faculdade de Ciências da Saúde, Departamento de Odontologia, Brasília, Brasil.

**Keywords:** Dental caries, Root caries, Collagenases, Microbial collagenase, Matrix metalloproteinases

## Abstract

Conventional views associate microbial biofilm with demineralization in root caries (RC) onset, while research on their collagenases role in the breakdown of collagen matrix has been sporadically developed, primarily in vitro. Recent discoveries, however, reveal proteolytic bacteria enrichment, specially *Porphyromonas* and other periodontitis-associated bacteria in subgingivally extended lesions, suggesting a potential role in RC by the catabolism of dentin organic matrix. Moreover, genes encoding proteases and bacterial collagenases, including the U32 family collagenases, were found to be overexpressed in both coronal and root dentinal caries. Despite these advancements, to prove microbial collagenolytic proteases’ definitive role in RC remains a significant challenge. A more thorough investigation is warranted to explore the potential of anti-collagenolytic agents in modulating biofilm metabolic processes or inhibiting/reducing the size of RC lesions. Prospective treatments targeting collagenases and promoting biomodification through collagen fibril cross-linking show promise for RC prevention and management. However, these studies are currently in the in vitro phase, necessitating additional research to translate findings into clinical applications. This is a comprehensive state-of-the-art review aimed to explore contributing factors to the formation of RC lesions, particularly focusing on collagen degradation in root tissues by microbial collagenases.

## Introduction

Root caries (RC) is an incident condition linked to life expectancy global increase and edentulism concurrent decline, which is attributed to improved hygiene standards and widespread access to fluoride products.^1-3^In the clinical scenario, challenges related to operational difficulties in restorative treatments of RC lesions arise due to microanatomical factors. These include issues such as moisture control, contamination with blood or gingival crevicular fluid, high mechanical stress concentration in the area, and cavities broad and shallow shape, which tends to spread horizontally.^4,5^ Additionally, significant amounts of organic material on the surface indicate that these lesions protocol often differs from the one applied to coronal surfaces.^4,6,7^ The available evidence regarding the most effective approaches for disease treatment is characterized by low to moderate certainty.^8-10^ Consequently, there is a need for studying new products and protocols in prevention and treatment of RC lesions.

Understanding RC etiopathogenesis can pave the way for innovative research, identifying potential therapeutics. Novel targets for these approaches can be found within dysbiotic biofilms. For example, recently identified overexpressed genes in RC-associated biofilms have emerged as potential drug targets, including those encoding bacterial collagenases, mobile elements, transcriptional regulators, carbohydrate metabolism enzymes, metabolic activity proteins, sugar transporters, stress tolerance factors, and pH regulators.^11-14^ Given these genes functions, to explore microbial collagenases involvement in the development of RC lesions seems to be particularly promising for developing future biochemical products that aim to preserve the integrity of the root collagen matrix. However, studies in this area face significant challenges. A recent systematic review showed only four studies examining microbial collagenases in clinical samples, either their gene expression or their activity.^15^ While bacteria would typically not expend energy to express a gene if it was nonfunctional to the cell, gene expression alone does not guarantee enzyme activity. A more thorough investigation is warranted to explore bacterial collagenolytic activity and enzyme inhibitors potential to modulate biofilm metabolic processes or to inhibit/reduce the size of RC lesions. If confirmed, the biofilm modulation could serve as complementary elements in future RC management in combination with fluoride products.

This state-of-the-art review aims to explore characteristics involved in RC lesions etiopathogenesis, particularly focusing on root collagen degradation by microbial collagenases. We revisited the peculiarities of dental root surfaces, lesion development, and other proteases part in collagen degradation. Finally, we discussed future perspectives for clinically preventing and managing RC.

### Revisiting the particularities of dental root surfaces composition and collagen structure

The cement covering root surfaces is characterized by a highly fibrous matrix comprising well-oriented collagen fiber bundles, which serve as anchorage points for periodontal ligament. It consists of approximately 45%–50% inorganic content and 50% organic content. Similarly, root dentin has high organic content (approximately 18%), with other components including 70% inorganic content and 12% water.^4,16^ The microstructure of the root dentin matrix contains tubules which accommodate odontoblasts cytoplasmic extensions (see Goldberg, et al.^16^ and Bosshardt, et al.^17^ for a comprehensive description of root hard tissues microstructure). In both root hard tissues, an elevated magnesium concentration can enhance hydroxyapatite crystals solubility compared to those found in enamel,^18^ since magnesium inhibit and regulate crystal growth by replacing calcium ions.^19,20^ Meanwhile, their organic matrix are primarily composed of type I collagen,^21^ although other non-collagen proteins, such as bone sialoproteins and osteopontin are also present in lower abundance.^17,21,22^

The term “collagen” encompasses 28 proteins, varying in size, function, and tissue distribution (Table 1) (for additional information, consult studies ^23-25^). They share a common characteristic: the formation of a supramolecular structure with a triple helix composed of three alpha polypeptide chains within an extracellular matrix.^24,26^ They are classified according to structure complexity, splice variants, presence of non-helical domains, and their assembly and function. Collagen can be either homotrimer (when formed by three identical chains) or heterotrimer (when formed by two or more different chains).^26^ In both cases, the three chains supercoil around the central axis, forming an extended helix. Each chain is formed by groups of 18 amino acids.^26,27^ A structural prerequisite for mounting on a triple helix is a glycine residue (always positioned in the center, the smallest amino acid) in each third position of the polypeptide chains, resulting in a Gly-X-Y repeat structure that characterizes and identifies the “collagen” domains. The X and Y positions are often occupied by proline and hydroxyproline.^24^

**Table 1:** Diversity class, genes, and tissue distribution of collagen types, adapted from Shoulders and Raines (2009), Ricard-Blum (2005) and Ruggiero and Ricard-Blum (2011)23-25

Type	Class	Gene	Distribution
I	Fibrillar	COL1A1, COL1A2	Ubiquitous and widespread: collagen found in the dermis, bone, tendon, ligament, and dentin
II	Fibrillar	COL2A1 (A, B)	Cartilage, vitreous
III	Fibrillar	COL3A3	Skin, blood vessels, intestine
IV	Network	COL4A1, COL4A2, COL4A3, COL4A4, COL4A5, COL4A6	Basement membranes
V	Fibrillar	COL5A1, COL5A2, COL5A3	Widespread: bone, dermis, cornea, placenta
VI	Network	COL6A1, COL6A2, COL6A3, COL6A4, COL6A5, COL6A6	Widespread: bone, cartilage, cornea, dermis
VII	Anchoring fibrils	COL7A1	Dermis, bladder
VIII	Network	COL8A1, COL8A2	Widespread: dermis, brain, heart, kidney
IX	FACIT	COL9A1, COL9A2, COL9A3	Cartilage, cornea, vitreous
X	Network	COL10A1	Cartilage
XI	Fibrilar	COL11A1(A, B, C), COL11A2, COL2A1	Cartilage, intervertebral disc
XII	Facit	COL12A1	Dermis, tendon
XIII	MACIT	COL13A1	Endothelial cells, dermis, eye, heart
XIV	FACIT	COL14A1	Widespread: bone, dermis, cartilage
XV	Multiplexin	COL15A1	Capillaries, testis, kidney, heart
XVI	FACIT	COL16A1	Dermis, kidney
XVII	MACIT Multiplexin	COL17A1	Hemidesmosomes in epithelia
XVIII	FACIT	COL18A1	Basement membrane, liver
XIX	FACIT	COL19A1	Basement membrane
XX		COL20A1	Cornea
XXI	FACIT	COL21A1	Stomach, kidney
XXII	FACIT	COL22A1	Tissue junctions
XXIII	MACIT	COL23A1	Heart, retina
XXIV	Fibrillar	COL24A1	Bone, cornea
XXV	MACIT	COL25A1	Brain, heart, testis
XXVI	FACIT	COL26A1	Testis, ovarys
XXVII	Fibrillar	COL27A1	Cartilage
XXVIII	------	COL28A1	Dermis, sciatic nerve

FACIT=fibrillar associated collagens with interrupted triple helix; MACIT=membrane-associated collagen with interrupted triple helix

**Figure 1:**
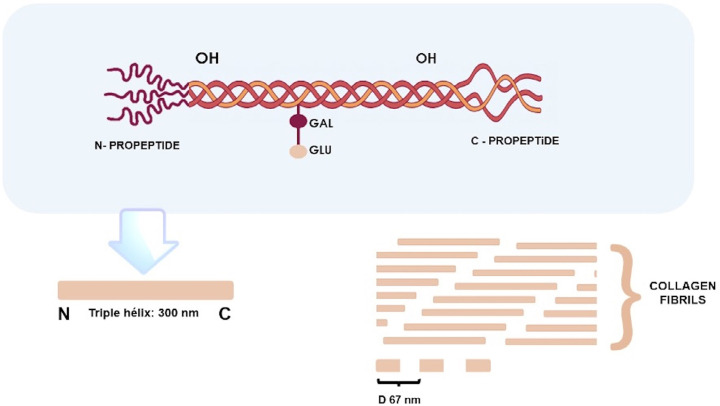
Representation of type I collagen adapted from Gelse, et al. (2003) and Varma, et al. (2016). N=N-terminal telopeptide region with 16 aa residues. C=C-terminal telopeptide region with 26 aa residues D=repeating unit of collagen fibril of length 67mm

The complex molecular structure of type I collagen, predominantly found on tooth root surfaces, is characterized by its ability to gather in oriented supramolecular aggregates with heterotrimeric structure, which contributes to molecular stabilization and dentin mechanical properties. Its fibrils represent a structural pillar and are perpendicularly connected by non-collagen proteins.^27,28^ This molecular structure is quite complex, formed by a triple helix with approximately 300 nm, comprised of three parallel polypeptide chains coiled together to form fibrils (Figure 1). By this arrangement, the N terminals of two axially adjacent triple helices are separated by the distance of D = 67 nm, and the N terminals of the two triple helices adjacent to the side are axially separated by 0.54 nm.^27^ This staggered arrangement creates alternating regions of low and high protein density along the fibril axis with a repetitive unit of length D (67 nm).^27,28^ For its characteristics, collagen can only be degraded by collagenases. In the aqueous phase, the triple helix is cleaved in its internal structure by digesting the amino group in a ‘Gly-Leu’ bond, enabling intramolecular flexibility, and facilitating specific proteolytic cleavage.^26,29^

**Figure 2:**
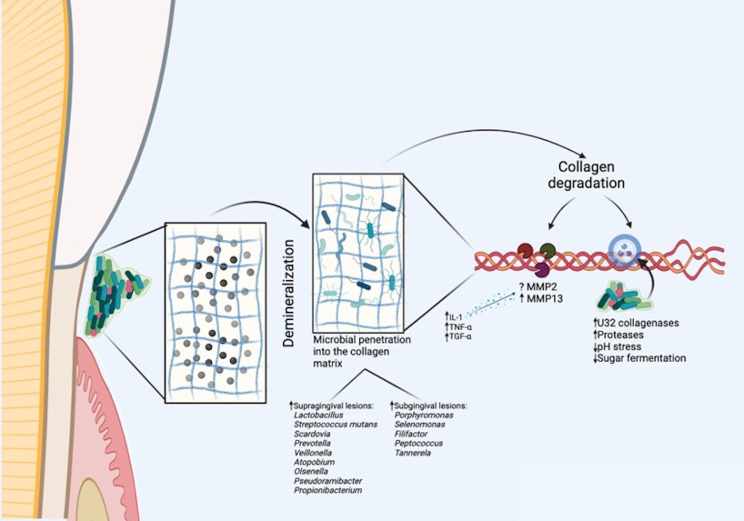
Representation of collagenases action on the root surface in a dysbiotic environment: upon root surface exposure, rapid demineralization is expected. This demineralization exposes root collagen fibers, which become accessible to microbial biofilm and their acidic metabolites. With the presence of growth factors and cytokines, these metabolites release and activate host matrix metalloproteases (MMPs). The high abundance of bacteria with their own collagenases in this niche, coupled with the expression of genes encoding microbial collagenases in root caries biofilms, suggests collaborative collagen degradation by the biofilm and MMPs

Dentinal collagen structure significance becomes apparent in the context of gingival recession. When recession occurs, a new ecological microenvironment emerges on the root surface, transitioning from an anaerobic to an aerobic setting with variable nutrient availability.^30^ The exposed root region, now susceptible to microbial infiltration, is vulnerable to demineralization by the acidic oral microbiota during the carious process.^29^ In addition, improper brushing of teeth or periodontal treatment itself can often damage or remove cement, rapidly exposing the dentin. This underscores the intricate interplay between root tissues composition and root caries development (Figure 2). Furthermore, due to these unique characteristics, the biomechanical conditions in this area are compromised. A maxillary premolar affected by RC examined with 3D-Finite Element Analysis revealed substantial stress concentration within the carious lesion. This suggests that the cavity resulting from RC may contribute to mechanical stress concentration, thereby influencing lesion development.^5^

### The two phases of the RC lesion development: demineralization and collagen degradation

The characteristics of biofilms on root surfaces has been extensively reviewed elsewhere.^31^ The first ecological concept of caries was proposed by Marsh (1994) and later extended by Takahashi and Nyvad.^29,32^ These hypotheses underscore that the enrichment of certain species in the oral microbiota, formerly viewed as odontopathogens, occurs in response to environmental changes induced by high consumption of fermentable carbohydrates. In other words, dental caries is not caused by a predetermined set of microorganisms but by composition alterations driven by external factors that shift microbiota balance toward demineralization.^29,33^ Regardless of the differences in the teeth crown and root substrates, dental caries initiates on both surfaces due to microbiota imbalance.

There are two successive phases of both coronal dentin and root surfaces lesion development, in which a proteolytic stage occurs after a demineralization stage.^29,33^ While demineralization can be reversed, the second stage of collagen degradation is irreversible. These two phases were shown on ultrastructural studies revealing that in early stages, a pH gradient from the outer surface dissolves minerals, maintaining the original cross-links between collagen fibers.^34,35^ Demineralized collagen serves as a support for colonizing bacteria (Figure 2). However, in contrast to coronal surfaces, root dentin does not require complete demineralization for bacterial colonization, as the channels of Sharpey’s fibers are possible colonization niches. In more advanced stages of RC lesion development, proteolytic enzymes degrades the exposed collagen, causing its fibers to lose their structural characteristics.^29^ However, a recent study suggested that cross-links between collagen bands may be degraded during demineralization,^36^ in which an exposed region of the collagen molecule is degraded by the activity of host-derived collagenolytic proteases (matrix metalloproteinases—MMPs).

MMPs are an important group of enzymes responsible for extracellular matrix degradation and are strongly related to physiological and pathological oral processes. A recent systematic review showed similar presence of MMP-2 in sound and carious root surfaces, while MMP-13 may be increased in root when compared to coronal carious dentin.^15^ The release and production of MMPs occur through cells, such as keratinocytes, polymorphonuclear leukocytes, macrophages, monocytes, fibroblasts, and mesenchymal cells. In the presence of growth factors and cytokines (interleukin-1, TNF-α, and TGF-α), these cells release MMPs into the extracellular environment.^37^MMPs are present in dental biofilms, gingival crevicular fluid, and saliva. When dental root is uncovered, bacteria and their acidic metabolites within the exposed collagen fibers can activate MMPs. Bacterial enzymes may be responsible for positive regulation of interleukin-1 present in gingival crevicular fluid and saliva. Interleukin is responsible for stimulating polymorphonuclear leukocytes and macrophages to release MMPs.^38^

### Are microbes involved in root caries collagen degradation?

In addition to MMPs, some well-known oral bacteria involved in oral diseases produce collagenases that could breakdown dentinal collagen (Figure 2). According to Takahashi and Nyvad,^29^ it is still questionable whether bacteria play a role in the initial stages of teeth organic components degradation. The mechanisms underlying insoluble collagen degradation in overall host tissues by bacterial collagenolytic proteases remain largely unexplored, especially referring to dentinal collagen. However, there is evidence indicating movement towards the collagen N-terminus by *C. histolyticum* type I collagenase (ColG) along collagen fibrils from rats tails resulting in complete fibril degradation within 20–30 minutes.^39^ This process depends on a catalytic zinc (Zn) ion, and it is promising in terms of Zn inhibition to stop bacterial collagenolytic activity in these tissues.

Bacterial collagenases are proteinases designed to target collagen, most relying on Zn for enzymatic function. They may possess certain capability to degrade collagen within host organisms,^40^ with examples including metalloproteases (M9 family) and serine proteases.^41^ All *Clostridium histolyticum* collagenases are members of the M9B subfamily (EC: 3.4.24.3) ^41^, while the *Porphyromonas* and other oral bacteria present U32 family collagenases (EC: 3.4.24.40).^12^ As outlined by Zhang, et al.^41^ (٢٠١٥), we adopt the term “bacterial collagenolytic proteases,” which encompasses both bacterial collagenases—enzymes that cleave helical regions of fibrillar collagen molecules under physiological conditions—and bacterial proteases that exclusively hydrolyze denatured collagen or type IV collagen. Therefore, “bacterial collagenolytic proteases” encompasses true bacterial collagenases as well as other proteases exhibiting collagenase activity, although most of them have never been described in oral microorganisms.^41^ Microbial collagenases and MMPs display unique characteristics that could lead to diverse effects on dentinal collagen matrix. Unlike mammalian collagenases, which cleave collagen at a single site, bacterial collagenase from M9 family carries out multiple cleavages ^42-44^ and more efficiently than MMPs.^45^Table 2 describes the classification of bacterial collagenolytic proteases. For more details on their diversity and characteristics, such as molecular mass (kDa) and action mechanisms, see Zhang, et al. ^41^ (2015).

**Table 2:** Classification of the predominant bacterial collagenases, adapted from Zhang, et al.41

Subgroup	Name	Representative microorganism	Substrate	Collagenolytic mechanism
U32	PrtC	*Porphyromonas gingivalis*^58^	Reconstituted type I collagen	Unknown catalytic type
		Analogue genes identified in several oral bacteria^12^	Heat-denatured type I collagen	
			Azocoll	
			FALGPA	
S1	SOT	*Streptomyces omiyaensis*	Type I, IV collagen	Serine proteases
S1	SGT	*Streptomyces griseus*	Type I collagen	
S8	MO-1	*Geobacillus collagenovorans*	Type I, IV collagen	Collagen-biding and fiber disassembly
S8	MCP -01	*Pseudoalteromonas sp.*	Type I, II, IV collagen, gelatin, casein, Pz peptide	
S8	Myroicolsin	*Myroides profundi*	Type I collagen, gelatin	
S53	Kumano lisin - As	*Alicyclobacillus sendaiensis*	Gelatin, relaxed collagen	Acts on denatured collagen at low pH and high temperature
M9B, CLASS I	ColG	*Clostridium histolyticum*	Type I, II, III collagen	Zn-activated endopeptidases that hydrolyze native collagens into a mixture of small peptides
	ColA	*Clostridium perfringens*	Type I collagen, Pz peptide, azocoll	
M9B, CLASS II	ColH	*Clostridium histolyticum*	Type I, II, III collagen	
M9A, CLASS II	VMC	*Vibrio mimicus*	Type I, II, III collagen, gelatin, Cbz-GPLGP, Cbz-GPGGPA	Triple-helical collagen cleaving, similarly to MMPs in the initial step of degradation. They target a site located three-quarters from the N-terminus, hydrolyzing the preferred peptide bond Xaa-Gly
	PrtVp	*Vibrio parahaemolyticus*	Type I, II, III, IV collagen, FALGPA	
M9A, CLASS III	VAC	*Vibrio alginolyticus*	Gelatin, casein, collagen, synthetic substrate	
	VPPC	*Vibrio parahaemolyticus*	Type I collagen, gelatin, casein, Cbz–GPGGPA	
M9A, CLASS III	VPM	*Vibrio parahaemolyticus*	Type I, II, III, IV collagen, gelatin, casein, Cbz-GPGGPA	

The first sign of oral bacteria involvement in RC can be a grand isolation of bacteria with collagenolytic proteases from root lesions, such as *Prevotella* and *Propionibacterium,* although not directly linked to their role in dentinal collagen degradation.^40,46-48^ Interestingly, a recent study showed that the bacterial composition of RC lesions located under the gingival margin is likely to have periodontal pathobionts: *Porphyromonas, Selenomonas, Filifactor, Peptococcus* and *Tannerela* inhabit RC lesions that extend beyond gingival margin.^49^ This suggests that microbiome in RC lesions expanding across the gingival margin would show an increase in bacterial proteolytic diversity. The expression of *P. gingivalis* collagenases-related genes in RC lesions has also been demonstrated.^12^ The integrated ecological hypothesis for caries and periodontitis ^50^ points to a common risk factor for both diseases, which are originated in the dynamic stability stage and emerges in response to nutritional imbalances in microbiota. According to authors of the integrated hypothesis, when inflammatory nutrients are intense and prolonged, the microbiota pH can move from dynamic stability to stages of proteolytic degradation, whereas the more intense and prolonged the episodes of exposure to dietary carbohydrates, the greater the pH shift to acidogenic and aciduric stages associated with caries.^50^ Given its physical connection with periodontal tissues, RC likely occupies an intermediate stage between acidogenic and proteolytic stages, harboring a diverse array of saccharolytic, aciduric, acidogenic, and proteolytic organisms.^31^ Additionally, microbes within RC can generate acid or ammonia through the catabolism of nitrogenous substrates, whether obtained exogenously or derived from the organic matrix of dentin.^11,31,51^

Microorganism significance and oral environment impact on dentin matrix collagen were investigated by van Strijp and colleagues through a series of *in situ* studies. These studies assessed the extent of denatured collagen following an experimental period under various cariogenic conditions.^52-55^ For this purpose, completely demineralized dentin specimens were positioned on the buccal surfaces of partial dentures worn by volunteers. Following an intraoral period of seven weeks, dentin samples were analyzed for denatured collagen. Intra-individual and inter-individual differences in collagen loss were found, likely attributed to microbiota composition differences, which colonized the demineralized specimens. In addition, differences in microorganisms ability to degrade the collagen matrix were credited for deviations in collagen loss.^52^ Gelatinolytic activity of isolated strains related to dentin matrix degradation was evaluated after identifying the colonizing microbiota of decalcified dentinal matrix. The predominant species found were *Streptococcus mitis, Peptostreptococcus spp.*, *Lactobacillus casei*, *Propionibacterium* species, and *Veillonella parvula*. Although no correlation was found with the severity of dentin matrix degradation, some gelatinolytic activity was observed in both saliva and dentin collagen.^55^ However, no correlation was observed between enzyme activity levels and collagen loss in dentin specimens.^53^

After these studies developed by van Strijp, et al., little has been published regarding oral bacteria involvement in root collagen degradation. More recently, few molecular studies have contributed to researching the role of microorganisms and their proteases in the progression of caries. Simon-Sóro, et al.^38^ (٢٠١٣) proposed a “tissue-dependent hypothesis” of caries by comparing the metagenomics of carious biofilms from enamel and dentin caries. The study showed different metabolic events occurring in each carious tissue, in which genes involved in acid stress tolerance and dietary sugar fermentation were overexpressed at the enamel caries biofilms, whereas collagenases and proteases were overexpressed in dentin cavities.^38^ Interestingly, genes for fermenting sugar in the diet and pH stress appeared at very low levels in dentin lesions, presenting an overrepresentation of genes involved in monosaccharides and disaccharides metabolism. Regarding deep dentin samples, genes related to host immune response were also overrepresented.^38^

Additional microorganisms also exhibited overexpression of genes associated with collagenases in RC, indicating their potential contribution to protein degradation, such as *Veillonella parvula* DSM 2008 (VPAR_RS05935 and VPAR_RS05390), *Veillonella dispar* ATCC 17748 (VEIDISOL_RS04770), and *Leptotrichia buccalis* (LEBU_RS05040). Those genes encode the collagenase-type protease family PrtC of the peptidase family U32.^56^ The U32 family of peptidases is a broad family of enzymes with little known structure and catalytic mechanism. This family presence has also been described in other pathogenic bacteria such as *P. gingivalis, Proteus mirabilis*, *Helicobacter pylori*, and *Aeromonas veronii.* In all cases, these bacteria hold putative collagenases, which are generally related to bacterial infections.^12,57^ For example, studies report the potential role of U32 collagenase in *P. gingivalis* virulence, which can degrade soluble fibrillar collagen type I and reconstitute at or below body temperature.^41^ A purified protease characterization, expressed from the bacterium’s *prtC* gene, enabled these analyses .^58^ PrtC peptidase has been documented to breakdown soluble and reconstituted fibrillar type I collagen, as well as heat-denatured type I collagen and azocoll.^58^ PrtC inhibitors include ethylenediaminetetraacetic acid (EDTA), thiol-blocking agents, and salivary histatin.^58^

Overexpression of putative *prtC* genes in *S. mutans* (SMU_761 and SMU_759 - *S. mutans* UA159) were also related to proteolytic activity and collagenases in RC lesions.^12^ SMU_761 encodes a 428 aa protein, whereas SMU_759 encodes a 308 aa protein. These findings are relevant due to this organism’s higher abundance and activity in RC lesions.^13^ However, as previously stated, gene expression alone does not ensure enzyme activity. When investigating enzymatic activity, some studies contradict the theory of bacterial collagenases involvement in RC. One study suggested that bacterial collagenases are highly sensitive to pH and unable to withstand acidic conditions (pH 4.3) during the dental demineralization stage, such as demonstrated for MMPs, not playing a substantial role to the development of carious lesions.^59^ Consistent with this, Tjaderhane, et al.^60^ (2015) observed no gelatinolytic or collagenolytic activity in bacterial samples and concluded its inactivity in dentinal caries. Although these results demonstrated the pH-dependent activation mechanism of human MMPs, they did not provide evidence for a similar mechanism in bacterial enzymes. Nevertheless, our current results contradict their findings. We showed no differences in *S. mutans* activity when using the synthetic collagen FALGPA (N-(3-[2-Furyl]acryloyl)-Leu-Gly-Pro-Ala) at acidic and neutral pH levels. This indicates that bacteria could exert some collagenolytic function even in lower pH environments, suggesting an intrinsic collagenolytic capability of *S. mutans* (unpublished data).

Besides, there is some evidence on *S. mutans* collagenolytic activity, such as its capacity to degrade collagen from rodent tendons.^61^ Still, another study showed that the same strain of *S. mutans* GS-5 collected from human carious lesions was able not only to induce caries in rodents, but also to promote bone tissue degradation, reinforcing the microbial role hypothesis in RC collagen degradation.^61^ Although studies have claimed that *S. mutans* constitutes only a small proportion of the microbiota,^62^ this microorganism is strongly related to the disease. In addition it is present at higher frequencies on decayed root surfaces than on biofilms on root surfaces ^63,64^ and can play an important role in RC progression.^13,31,63^ Yet, these results remain inconclusive, largely because of methodological challenges. For example, we have been striving to provide a more comprehensive description of *S. mutans* collagenase activity. Despite attempts to knockout SMU_761 and SMU_759 using various methods and strategies, the transformation proved to be inefficient.^65^ Although FALGPA and gelatin substrates exhibited degradation, we observed only minimal activity against type I collagen (unpublished data). It is crucial to note that results do not consider the natural composition of dental biofilms, which inherently comprise a diverse and multifunctional microbiota. Biofilm components assume different functions compared to when they are isolated, due to these communities multidimensional and highly complex nature.^46^ This indicates that studying isolates may yield different results in terms of collagenolytic activity compared to natural biofilms.

To summarize, so far we can state that host-derived MMPs initiates partial breakdown of the telopeptide region of dentin collagen molecules. Regarding bacterial collagenolytic proteases, there is controversy regarding the scientific evidence for their activity in carious lesions, alongside a notably small number of clinical studies.^15^ Nevertheless, U32 bacterial collagenases can hypothetically cleave collagen at multiple sites at the same time. Then, collagen molecules, now solubilized, could be irreversibly denatured under acidic conditions and body temperature. The presence of proteolytic bacteria and the activation of genes encoding collagenolytic proteases, along with the ones encoding collagen-binding proteins for highly active bacteria in RC,^11^ indicates potential contribution to matrix degradation.

### Future perspectives for translational research in prevention and management of root caries

A significant challenge in addressing RC lies in its clinical treatment. Invasive treatments pose various operational difficulties, including challenges in moisture control particularly in subgingivally extended lesions.^66^ Promoting remineralization is an important strategy for RC control.^67-69^ Non-invasive therapies with high concentrations of fluoride, such as 5000 ppm/F toothpastes and silver diamine fluoride, have a good body of evidence on controlling lesion progression and preventing new lesions.^70^ However, current studies emphasize collagen integrity preservation as a potential strategy for new adjunctive treatments in RC.

There is very limited clinical research on the use of collagenase inhibitors in root surfaces. For instance, the application of 2% chlorhexidine after acid-etching in restorative procedures has not demonstrated any significant effect in the longevity of non-carious root lesions.^71,72^ However, these studies utilized short follow-up periods and did not provide information on collagen stabilization in exposed roots. Furthermore, in both root and coronal caries, most investigated adjunctive therapeutic agents function as antimicrobials so far. Due to the inherent dysbiotic nature in RC etiopathogenesis, antimicrobials are ineffective in caries control, therefore its clinical application is meaningless. However, substances with phytochemical effects on dental tissue or the ability to modulate biofilm metabolic processes present some potential.^73-75^ Studies have investigated extracts abundant in phenols and polyphenols to assess their impact on the interplay of cross-linking, resistance to digestion, the activities of MMPs and other collagenases.^76^ Also, once the participation of U32 proteases in RC is confirmed, inhibition using metal ion-chelating substances like Zn and Fe^2^ could be considered. For instance, a hemi-synthetic anacardic acid compound derived from cashew nut shell liquid was capable of inhibiting about 90% of the *P. gingivalis* collagenase activity *in vitro*, probably due to its selective chelation of Fe^2^.^77^

Another adjunctive treatment option to prevent collagen breakdown involves the biomodification of dental root surface. Dentin biomodification strategy is based on the formation of inter- and intramolecular cross-links, which result in stabilization of the collagen constituting dentin organic matrix. Extensive *in vitro* studies have explored the capacity of various substances to promote cross-links in root collagen, which could reduce dentin matrix degradation and increase the biomechanical properties of healthy tissue.^78,79^ Several agents have been recognized by studies as cross-linkers, including glutaraldehyde (GA), formaldehyde, carbodiimide and epoxy compounds.^76,78,80-82^ However, adverse effects such as toxicity and/or instability limited these substances use *in vivo*. To the best of our knowledge, no relevant clinical data is currently available to either prevent or impede RC lesions.

Recently, natural agents available in fruits, bark, leaves, and seeds, have been considered biocompatible and stable for a long period of time in animals. Natural agents are characterized by lower toxicity compared to synthetics and can be considered renewable. Most of these agents derive from plants and are their secondary metabolites. Polyphenols, for example, can be subdivided into phenolic acids, flavonoids, stilbenes, and lignans. These agents are capable of forming cross-links through covalent bonds and hydrogen bonds.^83^ Examples include proanthocyanidins (PAC), genipin, and cranberry .^76^ Promising results have shown that PAC could efficiently stabilize collagen by increasing its resistance against caries *in vitro* under artificial lesion formation.^78^ Similarly, other study used an *in vitro* pH cycling model to evaluate the effect of GSE (grape seed extract that contains PAC) on artificial root caries remineralization, showing a positive effect in demineralization and/or remineralization processes.^84^ Nevertheless, it is important to bear in mind that structure modifications using cross-linking agents could potentially involve not only collagen stabilization but also remineralization effects.

It is crucial to note that the positive outcomes of anti-collagenases and cross-linkers are currently derived from *in vitro* experiments that simulate demineralizing conditions using cyclic pH models. While these conditions pose challenges for immediate clinical applicability, if these treatments effectiveness are confirmed, they might represent low-cost and easy-to-apply adjunctive non-invasive therapies for controlling RC lesions.

## Conclusion

Root caries lesion formation is likely a two-stage process, where collagen breakdown follows mineral loss. MMPs presence, particularly MMP-13, must be associated with collagen denaturation in RC. Given the prevalence of proteolytic bacteria in RC-associated biofilms, their contribution to the second stage of lesion formation is plausible. Nevertheless, proving the role of microbial collagenases in RC remains a significant challenge. A more in-depth investigation into microbial collagenases role in these lesions is warranted. Modulating biofilm by inhibiting collagenases or protecting the collagen matrix from degradation by using cross-linking agents could be a targeted approach for RC treatments and prevention.
